# Caregivers’ Attitudes Toward Baby-Led Weaning: Development and Psychometric Validation of the BLW-CAS

**DOI:** 10.3390/healthcare14142214

**Published:** 2026-07-21

**Authors:** Rita Rocío Márquez-Díaz, María Dolores Ruiz-Fernández, María Isabel Ventura-Miranda, Isabel María Fernández-Medina

**Affiliations:** 1 Hospital Universitario Virgen de las Nieves, 18014 Granada, Spain; rmd377@inlumine.ual.es; 2 Department of Nursing Science, Physiotherapy and Medicine, University of Almería, 04120 Almería, Spain; mvm737@ual.es (M.I.V.-M.); isabel_medina@ual.es (I.M.F.-M.); 3 Facultad de Ciencias de la Salud, Universidad Autónoma de Chile, Temuco 4780000, Chile

**Keywords:** baby-led weaning, complementary feeding, caregivers, attitudes, psychometrics, scale development

## Abstract

**Highlights:**

**What are the main findings?**

- The BLW-CAS was developed and psychometrically validated among Spanish caregivers.
- The final 16-item scale showed acceptable-to-excellent internal consistency across subscales and good test–retest reliability.

**What are the implications of the main findings?**

- A three-factor structure captured predisposition toward BLW, feeding-related self-efficacy, and practical skills for its implementation.
- The BLW-CAS may support research and the assessment of caregiver attitudes toward BLW in complementary feeding.

**Abstract:**

**Background/Objectives:** Baby-Led Weaning (BLW) is an increasingly adopted complementary feeding approach in which infants self-feed family foods rather than being spoon-fed purées. Caregivers’ attitudes play a central role in its adoption and implementation, yet no validated instrument has been specifically designed to assess caregivers’ attitudes toward BLW. This study aimed to develop and psychometrically validate the BLW Caregiver Attitudes Scale (BLW-CAS). **Methods:** A methodological psychometric study was conducted in two phases. Scale development involved a literature review, expert panel evaluation, and cognitive pretesting with caregivers. Psychometric validation was conducted among 256 caregivers, while temporal stability was assessed in an independent test–retest sample of 92 caregivers. Content validity, internal consistency, temporal stability, construct validity, and convergent validity were examined using established psychometric methods. **Results:** The final 16-item BLW-CAS comprised three dimensions: Predisposition, Self-efficacy, and Practical Skills. The revised scale achieved an S-CVI/Ave of 0.93. The three-factor exploratory solution accounted for 67.46% of the total item variance. The three-factor CFA model showed good overall fit (CFI = 0.995; TLI = 0.994; RMSEA = 0.052; SRMR = 0.066). Internal consistency was acceptable to excellent across subscales (α = 0.757–0.913; ω = 0.807–0.953), and test–retest reliability was good (ICC = 0.832). Positive small-to-moderate correlations with general self-efficacy (r = 0.298) and decision satisfaction (r = 0.350) supported convergent validity. **Conclusions:** The Spanish BLW-CAS is a concise, multidimensional instrument with promising psychometric properties for assessing caregivers’ attitudes toward BLW, supporting future research and the evaluation of interventions related to complementary feeding practices.

## 1. Introduction

The World Health Organization (WHO) and the United Nations Children’s Fund (UNICEF) recommend initiating breastfeeding within the first hour after birth and maintaining exclusive breastfeeding for the first six months of life [1]. After this period, infants’ nutritional requirements evolve and exceed what exclusive breastfeeding alone can provide. Consequently, the introduction of complementary foods becomes necessary. According to international guidelines, breastfeeding should continue alongside complementary foods up to two years of age or beyond [1].

Complementary feeding refers to the gradual introduction of foods and beverages other than breast milk into the infant’s diet [2]. Traditionally, this stage has been approached through spoon-feeding pureed foods prepared by combining one or more ingredients with milk, broth, or water [3,4]. However, growing evidence supports the use of alternative complementary feeding approaches, such as Baby-Led Weaning (BLW) [5,6,7].

BLW involves offering infants whole, appropriately prepared foods without crushing or grinding them, allowing infants to self-feed using their own motor abilities, such as grasping food with their hands [6,8,9,10]. Although chronological age is often used as a reference for initiating complementary feeding, BLW requires that infants reach specific neurodevelopmental milestones, including the ability to sit independently and coordinate hand-to-mouth movements, and no longer exhibit the extrusion reflex [11].

Through the BLW approach, infants determine which foods to eat, the amount consumed, and the pace of feeding, thereby adopting a more active role in the feeding process and participating in family meals, which may positively influence family mealtime dynamics [9]. BLW is considered a responsive feeding approach implemented during structured mealtimes, supporting infants’ ability to respond to hunger and satiety cues [12].

This feeding method has been associated with several potential benefits for both infants and caregivers. By promoting self-feeding with whole foods, BLW may support the development of essential motor skills, such as hand-eye coordination and chewing abilities, contributing to psychomotor development [13]. In addition, BLW may encourage healthier eating habits and foster a positive relationship with food by allowing infants to explore a variety of tastes, textures, and flavors at their own pace [4].

Nevertheless, potential risks associated with BLW must also be considered. One of the main concerns relates to the risk of choking, particularly during the early stages of complementary feeding [10]. Adequate caregiver knowledge regarding appropriate food selection, preparation techniques, and recognition of infant feeding cues is therefore essential to minimize this risk [10,11]. Concerns regarding nutritional adequacy have also been raised, especially when caregivers lack sufficient guidance on offering a balanced diet [12,14,15]. However, available evidence suggests no clear increase in choking risk or adverse effects on iron status among infants following BLW compared with traditional spoon-feeding, although the evidence remains limited [16]. Appropriate supervision and the inclusion of iron-rich foods in infants’ diets remain important [10,17].

Caregiver knowledge and perceptions play an important role in shaping complementary feeding practices. Evidence suggests that infant feeding education can influence caregivers’ feeding decisions and behaviors. In Spain, early studies reported limited awareness and use of BLW [18], whereas more recent cross-sectional data indicate high levels of familiarity with and recommendation of this approach [19]. These findings reflect the growing integration of BLW into contemporary childcare practices and highlight the need for tools that allow systematic assessment of caregivers’ attitudes toward this feeding method.

Although BLW is increasingly recognized as a viable alternative to traditional spoon-feeding approaches, most existing research has focused on caregivers’ knowledge, practices, perceptions, or experiences with this feeding method. In contrast, caregivers’ attitudes toward BLW remain relatively underexplored. In this study, caregivers’ orientation toward BLW is conceptualized within a broader attitudinal-readiness framework. Contemporary attitude theory defines attitude primarily as the favorable or unfavorable evaluation of a specific behavior, whereas behavioral models distinguish attitude from perceived behavioral control or self-efficacy [20,21,22]. Because BLW adoption may depend both on evaluative predisposition toward the method and on perceived capability to implement it, the present instrument was developed to capture these related but conceptually distinct components. Accordingly, the BLW-CAS assesses predisposition toward BLW together with feeding-related self-efficacy and perceived practical skills.

Despite their importance, there are currently no validated instruments specifically designed to assess caregivers’ attitudes toward BLW. The absence of standardized measurement tools limits the comparability of findings across studies and restricts the evaluation of educational or clinical interventions related to complementary feeding practices. Therefore, the aim of this study was to develop and psychometrically validate the BLW Caregiver Attitudes Scale (BLW-CAS) to assess caregivers’ attitudes toward the BLW complementary feeding approach. Specifically, the study examined its reliability, temporal stability, content validity, construct validity, and convergent validity.

## 2. Materials and Methods

The BLW-CAS was originally developed and psychometrically validated in Spanish. All psychometric analyses reported in this study were conducted using the Spanish version. The English wording included in this manuscript is provided solely for reporting purposes and should not be interpreted as an independently validated English-language version. The original Spanish version of the scale is available as Supplementary Material.

### 2.1. Design

A methodological psychometric study was conducted to develop and validate the BLW-CAS. The study comprised two sequential phases: scale development and content validation, followed by psychometric evaluation in a caregiver sample. The main validation phase was cross-sectional and included 256 caregivers. Temporal stability was assessed separately in a pilot test–retest sample of 92 caregivers before the large-scale validation phase. The study design and reporting were informed by relevant COSMIN methodological recommendations for instrument development and validation [23], while also considering relevant STROBE recommendations for the cross-sectional components [24].

### 2.2. Study Setting and Sampling

Participants were recruited by convenience sampling through primary care centers in southeastern Andalusia and by nationwide online dissemination through institutional channels. The target population comprised caregivers of children aged 0–24 months. The use of both recruitment strategies allowed access to caregivers through both clinical and online settings and facilitated self-administered participation.

For structural validity, the COSMIN Study Design Checklist recommends a sample of at least seven participants per item and at least 100 participants for factor analysis [23]. The final validation sample included 256 caregivers, exceeding this criterion for the original 19-item version of the scale.

### 2.3. Inclusion and Exclusion Criteria

Participants were eligible if they were aged 18 years or older, provided informed consent, and were the caregiver of a healthy, full-term child with age-appropriate psychomotor development. Exclusion criteria included significant difficulties in reading or writing, having a child born preterm, child health or developmental conditions requiring specialist medical care, and caregivers being unable to provide information due to health-related reasons.

### 2.4. Scale Development

The scale was developed on the basis of a literature review and an item generation process focused on caregivers’ attitudes toward BLW. Content validity was evaluated by an 11-member multidisciplinary expert panel comprising pediatric nurses, a midwife, pediatricians, a family physician, and a dietitian-nutritionist. Experts evaluated the relevance of each item using a four-point Likert scale ranging from “not at all relevant” to “very relevant.” Experts were also invited to provide qualitative feedback to improve clarity, wording, and conceptual relevance of the items. Following expert feedback, the item wording was revised and re-evaluated before cognitive pretesting.

Content validity was quantified using the Content Validity Index (CVI) [25,26]. The scale-level CVI was calculated as the average of the item-level CVI values (S-CVI/Ave), with values ≥0.90 considered indicative of adequate content validity.

A cognitive pretest was subsequently conducted with 12 caregivers to assess item clarity, comprehensibility, applicability, and questionnaire length. Feedback from the cognitive pretest informed wording refinement before large-scale administration. Temporal stability was then examined in 92 independent caregivers who completed the 19-item questionnaire twice, 15 days apart. Unique identifiers were used to match responses across both administrations. The questionnaire used a five-point Likert response scale ranging from 1 (strongly disagree) to 5 (strongly agree), with higher scores indicating more favorable attitudes toward BLW.

### 2.5. Psychometric Evaluation

#### 2.5.1. Reliability

Internal consistency was assessed using Cronbach’s alpha (α) and McDonald’s omega (ω). For the final scale and subscales, α was calculated from observed item scores and ω from the polychoric correlation matrix. For the preliminary 19-item version, ordinal α was additionally estimated from polychoric correlations, and conventional and standardized α values were reported for comparison. Because the retained solution was multidimensional, internal consistency was interpreted primarily at the subscale level, while total-score coefficients were reported descriptively. Values of 0.70 or higher were considered indicative of acceptable internal consistency. Temporal stability was assessed using the intraclass correlation coefficient (ICC; two-way mixed-effects model, absolute agreement, single measures) [27], with values above 0.75 interpreted as indicating good test–retest reliability. Agreement between test and retest scores was additionally examined using the Bland–Altman method [28].

#### 2.5.2. Construct Validity

Construct validity was assessed through exploratory factor analysis (EFA) and confirmatory factor analysis (CFA). The main EFA was performed in R on a polychoric correlation matrix [29] using principal axis factoring and oblimin rotation, given the expected correlation between latent factors. The Kaiser–Meyer–Olkin (KMO) statistic and Bartlett’s test of sphericity were calculated from the Pearson correlation matrix as factorability diagnostics. The number of factors to retain in the main polychoric EFA was informed by parallel analysis, examination of the scree plot, factor interpretability, and theoretical coherence. [30]. Items with low communalities or factor loadings below 0.40 were considered for removal during the factor analysis process.

CFA was subsequently used to preliminarily evaluate the fit of the three-factor structure identified in the EFA within the same validation sample. Because the items were ordinal, models were estimated using the weighted least squares mean and variance adjusted (WLSMV) estimator [31]. Model fit was evaluated using χ^2^, the Comparative Fit Index (CFI), the Tucker–Lewis Index (TLI), the Root Mean Square Error of Approximation (RMSEA), and the Standardized Root Mean Square Residual (SRMR).

#### 2.5.3. Convergent Validity

Convergent validity was assessed by examining associations between BLW-CAS total scores and two validated external measures theoretically related to caregivers’ attitudes toward BLW: the Spanish version of the General Self-Efficacy Scale [32], a 10-item measure of perceived competence in managing difficult situations, and the Spanish version of the Satisfaction With Decision Scale [33], a 6-item measure of satisfaction with a health-related decision. Higher scores indicated greater general self-efficacy and greater satisfaction with the feeding decision, respectively. Pearson correlation coefficients with 95% confidence intervals were calculated, and Holm correction was applied for multiple testing.

We hypothesized positive, small-to-moderate correlations between BLW-CAS scores and both general self-efficacy and decision-making satisfaction.

### 2.6. Data Collection

Literature review and initial item generation began in June 2023. Expert review and cognitive pretesting with caregivers were conducted after ethical approval, between July and September 2023. The large-scale psychometric validation took place between February and March 2024. The test–retest assessment was completed prior to the large-scale administration of the scale in an independent pilot sample.

Participants completed the questionnaire either online or in person using the same item wording and response options.

To enhance data quality and ensure response authenticity, online data collection used Google Forms, with one submission permitted per authenticated Gmail account. Email verification procedures and response timestamps were used to identify potential duplicate or atypical completion patterns. Data were systematically screened for inconsistencies and indicators of non-genuine or automated responses, and no such cases were identified.

All BLW-CAS items were mandatory in the online questionnaire, and in-person questionnaires were checked for completeness at the time of administration. Therefore, no item-level missing data were present for the scale items, and no imputation was required.

### 2.7. Data Analysis

Analyses were conducted using IBM SPSS Statistics version 27 (IBM Corp., Armonk, NY, USA) and R version 4.5.2 (R Foundation for Statistical Computing, Vienna, Austria) in RStudio version 2026.06.0 (Posit PBC, Boston, MA, USA). Continuous variables were summarized using means and standard deviations or medians and interquartile ranges, as appropriate, and categorical variables were described using frequencies and percentages.

In the independent pilot test–retest sample, paired comparisons, ICC estimates, and Bland–Altman plots were used to evaluate temporal stability. Because the final item set was determined after item reduction in the main validation sample, test–retest analyses were recalculated using only the retained items from the pilot test–retest dataset. In the validation sample, descriptive item statistics, internal consistency estimates, EFA, CFA, and correlations with external variables were computed. A two-sided *p* value < 0.05 was considered statistically significant.

### 2.8. Bias

The use of convenience sampling and recruitment through institutional and online channels may have introduced selection bias, favoring caregivers with greater interest in or prior experience with BLW. The relatively homogeneous sociodemographic profile of the sample may also limit representativeness. To reduce information bias, the scale was administered as a self-administered questionnaire under standardized instructions and refined through expert evaluation and cognitive pretesting. Eligibility criteria and additional online data quality controls were applied to minimize the risk of duplicate or non-genuine responses.

### 2.9. Ethical Considerations

The study was conducted in accordance with the Declaration of Helsinki and approved by the Comisión de Ética e Investigación del Departamento de Enfermería, Fisioterapia y Medicina de la Universidad de Almería (approval code: EFM 280/23; approval date: 21 July 2023). All participants provided informed consent before participation.

## 3. Results

Content validity analysis showed that the initial version achieved an S-CVI/Ave of 0.90. After item rewording based on expert feedback, the revised version showed an improved S-CVI/Ave of 0.93, supporting adequate content validity. The main item modifications are summarized in Table 1, and the expert panel is described in Table 2.

The cognitive pretest with 12 caregivers indicated good item clarity, adequate comprehensibility, and acceptable questionnaire length. Participants reported no major difficulties in completing the questionnaire, and completion time ranged from approximately 5 to 10 min.

**Figure 1:**
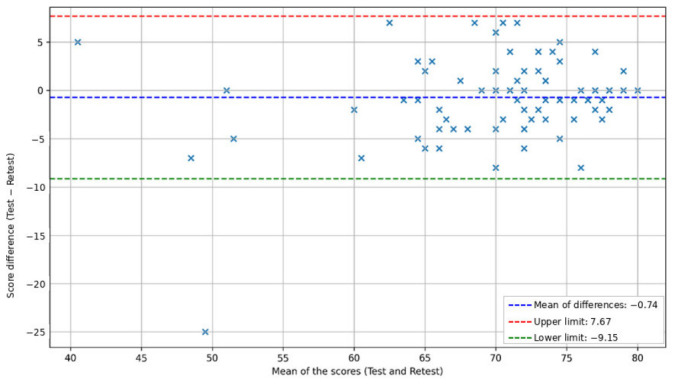
Bland–Altman plot for the test–retest reliability analysis of the BLW-CAS. Note: Each × represents one participant. The central line represents the mean difference (bias), and the upper and lower lines indicate the 95% limits of agreement (bias ± 1.96 × SD).

Temporal stability was examined in an independent pilot test–retest sample of 92 caregivers. Although the 19-item version was administered in this phase, test–retest analyses were recalculated after item reduction using the retained 16 items. Mean total scores were 70.54 (SD = 7.95) at test and 71.28 (SD = 6.94) at retest. The mean difference (test − retest) was −0.74 (SD = 4.29; 95% CI −1.63 to 0.15; *p* = 0.102). The ICC for absolute agreement was 0.832 (95% CI 0.757–0.886; *p* < 0.001), indicating good temporal stability of the final 16-item BLW-CAS. Bland–Altman analysis showed a mean bias of −0.74 with 95% limits of agreement from −9.15 to 7.67; 98.9% of observations fell within these limits (Table 3; Figure 1).

A total of 256 caregivers participated in the validation study. The sample was predominantly mothers (99.6%), with a mean age of 34.07 years (SD = 3.72). Most participants had university education (77.0%), lived in urban areas (82.4%), and reported prior experience with BLW (88.3%). Overall, attitudes toward BLW were favorable, with a mean item score of 4.38 (SD = 0.58) and a mean total score of 70.10 (SD = 9.25) for the final 16-item version. Across items, responses showed a marked tendency toward agreement, particularly for statements referring to the perceived benefits of BLW. Participant characteristics are presented in Table 4, and descriptive statistics for individual items are presented in Table 5.

**Figure 2:**
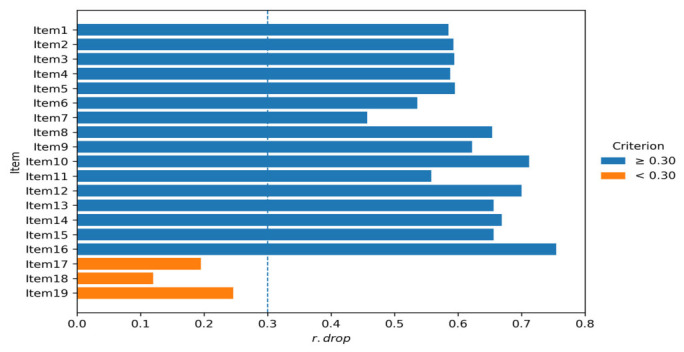
Corrected item-total correlations (r.drop) for the initial 19-item BLW-CAS. Note. The dashed vertical line indicates the minimum acceptable corrected item-total correlation threshold (r.drop = 0.30). Items below this threshold were considered for removal.

The initial 19-item version showed high internal consistency. Because the scale used ordered categorical response options, reliability was primarily estimated from polychoric correlations. Ordinal alpha was 0.932 and omega was 0.937, indicating high internal consistency. For comparison with previous literature, conventional Cronbach’s alpha was also computed, yielding α = 0.889 (standardized α = 0.908). Examination of corrected item-total correlations showed that items 17, 18, and 19 contributed weakly to the overall homogeneity of the scale (r.drop = 0.195, 0.120, and 0.246, respectively). Consistently, deletion of these items would have produced a slight increase in conventional alpha (Table 6; Figure 2).

Construct validity was first examined through exploratory factor analysis (EFA). The main EFA was conducted in R using a polychoric correlation matrix, principal axis factoring, and oblimin rotation. Using the Pearson correlation matrix for complementary factorability diagnostics, sampling adequacy for the initial 19-item solution was high (KMO = 0.903), and Bartlett’s test of sphericity was significant (χ^2^(171) = 2573.33, *p* < 0.001), supporting factorability. For the main polychoric EFA, factor loadings ≥0.40 were considered salient, and communalities <0.30 were considered indicative of poor item representation.

**Figure 3:**
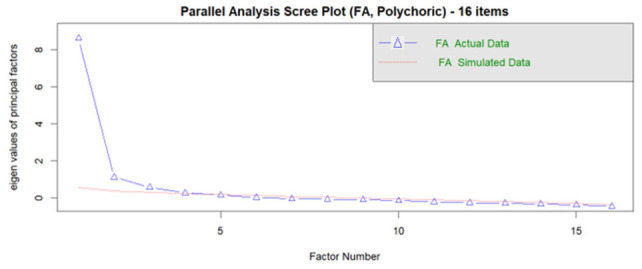
Parallel analysis based on polychoric correlations for the 16-item BLW-CAS.

In the preliminary 19-item polychoric EFA, items 17–19 showed very low communalities (h^2^ = 0.113–0.135), indicating poor representation in the latent structure (Table 7). Items 17 and 19 had maximum factor loadings below 0.40, whereas item 18 reached a loading of 0.423 but retained a very low communality (h^2^ = 0.135). These items were therefore removed, and the analysis was repeated with the remaining 16 items. For the refined 16-item set, the corresponding Pearson-based adequacy diagnostics were KMO = 0.908 and Bartlett’s χ^2^(120) = 2478.36 (*p* < 0.001). Parallel analysis based on the polychoric correlation matrix suggested a four-factor solution (Figure 3). However, inspection of the four-factor solution showed that the additional factor was poorly defined, had fewer than three salient loadings, and lacked theoretical coherence. Accordingly, a three-factor solution was retained on the basis of parsimony and interpretability.

The final three-factor structure accounted for 67.46% of total item variance (Table 8). The factors were interpreted as Predisposition, Self-efficacy, and Practical Skills. Factor correlations were positive and moderate in the EFA solution (Φ = 0.372–0.551), indicating that the dimensions were related but distinguishable. Item communalities ranged from 0.458 to 0.944, supporting adequate representation of the retained items (Table 9).

A small number of items showed modest secondary loadings in the EFA, particularly items 2, 4, 5, and 15; however, their primary loadings were higher and conceptually consistent with the retained factor assignments.

The Predisposition factor included eight items reflecting favorable beliefs and motivation toward BLW, including perceived benefits for infant autonomy, family meals, and positive relationships with food. The Self-efficacy factor comprised four items referring to caregivers’ confidence in making feeding-related decisions and recognizing their child’s hunger and satiety cues.

The Practical Skills factor included four items capturing perceived ability to implement BLW in everyday contexts, such as managing mess, acquiring feeding-related information, responding to choking situations, and adapting family meals.

Accordingly, the final 16-item BLW-CAS yields a total score ranging from 16 to 80, with higher scores indicating more favorable caregiver attitudes toward BLW. Subscale scores range from 8 to 40 for Predisposition and from 4 to 20 for Self-efficacy and Practical Skills. Mean item scores ranging from 1 to 5 may also be calculated for the total scale and each subscale to facilitate interpretation. Given the multidimensional structure, subscale scores should be interpreted alongside the total score, particularly when dimension-specific conclusions are of interest.

Internal consistency for the final 16-item version was acceptable to excellent across the three subscales. Cronbach’s alpha and omega were 0.913 and 0.953 for Predisposition, 0.830 and 0.883 for Self-efficacy, and 0.757 and 0.807 for Practical Skills, respectively. Total-score coefficients were α = 0.923 and ω = 0.948 and are reported descriptively given the multidimensional structure (Table 10). Correlations between subscales were moderate (Spearman’s rho = 0.510–0.585; all *p* < 0.001), supporting a multidimensional but coherent structure (Table 11).

The three-factor structure was subsequently evaluated using confirmatory factor analysis (CFA) with ordinal indicators and WLSMV estimation (*N* = 256). The model showed excellent incremental fit and acceptable absolute fit: χ^2^(101) = 170.37, CFI = 0.995, TLI = 0.994, RMSEA = 0.052 (95% CI [0.038, 0.065]), and SRMR = 0.066. All standardized factor loadings were moderate to high (λ = 0.591–0.943) (Table 12). Latent factor correlations ranged from 0.712 to 0.784, indicating substantial relatedness between the three dimensions. Although the unidimensional model showed a high CFI, it presented poorer absolute fit than the retained three-factor solution, particularly for RMSEA and SRMR (CFI = 0.980, TLI = 0.977, RMSEA = 0.104, SRMR = 0.099), providing preliminary support for the multidimensional structure of the scale.

As further evidence of construct validity, the total BLW-CAS score correlated positively with general self-efficacy (r = 0.298, 95% CI [0.182, 0.406], *p* < 0.001) and satisfaction with decision-making (r = 0.350, 95% CI [0.238, 0.453], *p* < 0.001), indicating small-to-moderate effect sizes. These associations remained significant after Holm correction and provide preliminary convergent evidence for the overall BLW-CAS score (Table 13).

## 4. Discussion

This study developed and conducted an initial psychometric evaluation of the BLW-CAS, a Spanish-language instrument designed to assess caregivers’ orientation toward the Baby-Led Weaning complementary feeding approach. The final 16-item version showed adequate content validity, good temporal stability, acceptable-to-excellent internal consistency across subscales, and a three-factor internal structure identified by EFA and preliminarily evaluated by CFA. Although the dimensions were interrelated, the three-factor model showed more favorable overall fit indices than a unidimensional alternative, supporting a multidimensional representation of the broader attitudinal-readiness construct assessed by the BLW-CAS. These findings are in line with expectations for early-stage instrument validation and provide preliminary support for the use of the BLW-CAS in research settings.

The sociodemographic profile of the sample is broadly consistent with patterns described in previous BLW research. Participants were predominantly mothers with higher educational attainment, urban residence, and prior experience with BLW. Similar profiles have been reported in studies suggesting that BLW is more frequently adopted among families with higher levels of education and access to information [18,34]. This profile should be considered when interpreting the generally favorable attitudes observed. Formal floor and ceiling effects were not quantified; however, the high mean scores of several items, particularly within the Predisposition domain, suggest possible ceiling effects and restricted response variability. This concentration of favorable responses may have influenced item performance and the observed factor solution and should be examined in more heterogeneous samples.

The BLW-CAS also differs from existing feeding-related instruments. The Child Feeding Questionnaire assesses parental beliefs, attitudes, and practices related to child feeding, with a particular focus on obesity proneness, whereas the Infant Feeding Style Questionnaire assesses feeding beliefs and behaviors across broader infant feeding styles [35,36]. Previous BLW-specific instruments have assessed parents’ perceptions of the method or the extent to which complementary feeding practices reflect a baby-led approach [37,38]. In contrast, the BLW-CAS specifically targets BLW-related attitudinal readiness by integrating evaluative predisposition toward the method with feeding-related self-efficacy and perceived practical skills. It should therefore be considered complementary to, rather than a replacement for existing BLW-specific and broader parental feeding measures.

A key finding is that the broader BLW-related attitudinal-readiness construct assessed by the BLW-CAS was represented by three related dimensions: Predisposition, Self-efficacy, and Practical Skills. The Predisposition dimension captured positive beliefs regarding infant autonomy, family meals, and the perceived benefits of BLW. The Self-efficacy dimension reflected caregivers’ confidence in feeding-related decision-making and in recognizing their child’s hunger and satiety cues. The Practical Skills dimension referred to the perceived ability to implement BLW in everyday contexts, including managing mess, acquiring feeding-related information, and adapting family meals. Confirmatory analyses indicated that Self-efficacy and Practical Skills were closely related but were retained as separate dimensions in the three-factor model.

This three-dimensional structure is broadly consistent with previous literature describing BLW as an approach characterized by greater infant autonomy, reduced caregiver control, and participation in family feeding contexts [39,40]. Within this broader framework, the coexistence of Predisposition, Self-efficacy, and Practical Skills is theoretically plausible. Endorsement of the perceived benefits of BLW may favor its adoption, whereas successful implementation may also require confidence in feeding-related decisions and competence in managing practical issues such as meal organization, mess, and safety concerns.

Three items originally intended to assess the social context of BLW were excluded from the final solution because they showed weak item-total correlations, low communalities, and unstable factor loadings. However, this exclusion should not be interpreted as evidence that social influences are irrelevant to BLW. Rather, in this sample and with the wording used, these items did not share sufficient common variance with the core construct assessed by the BLW-CAS. Previous research suggests that family views, professional advice, social support, and access to information may meaningfully shape caregivers’ experiences and influence the uptake of BLW [34,41,42,43]. Therefore, social-context influences may represent an adjacent contextual domain that could be assessed through a separate or more specifically developed instrument.

The item related to responding to choking situations deserves specific mention. In the EFA, this item had the lowest primary loading among the retained items (0.433; h^2^ = 0.470), although it remained above the prespecified salience threshold of 0.40 and showed adequate communality. Its standardized CFA loading was 0.730. Alternative models excluding this item were not explored because the item met the predefined retention criteria and was also considered conceptually and clinically relevant. Fear of choking is one of the most frequently reported concerns associated with BLW among caregivers and healthcare professionals and has been consistently highlighted in both reviews and empirical studies of parental and professional attitudes [11,41]. At the same time, recent evidence suggests that BLW does not appear to increase choking risk compared with traditional spoon-feeding when appropriate guidance is followed [11,16,44]. Its inclusion therefore enhances the clinical relevance and real-world applicability of the scale by capturing a concern that may meaningfully influence caregiver attitudes toward BLW.

The observed positive correlations with general self-efficacy and satisfaction with decision-making provided preliminary convergent evidence for the overall BLW-CAS score. Their modest magnitude suggests that the BLW-CAS captures a specific attitudinal domain related to complementary feeding rather than simply reflecting a broad sense of parental confidence or general decision satisfaction.

### Strengths and Limitations

This study has notable strengths, including a systematic scale-development process involving literature review, multidisciplinary expert evaluation, and cognitive pretesting with caregivers. In addition, temporal stability was assessed in an independent test–retest sample. Nevertheless, some limitations should be considered. The validation sample was relatively homogeneous and consisted mainly of mothers with higher education, urban residence, and prior familiarity with BLW, while convenience recruitment may have favored caregivers with greater interest in or more favorable attitudes toward this feeding approach. These characteristics may limit generalizability to more diverse caregiver populations. Furthermore, the factorial structure was explored and preliminarily evaluated within the same validation sample rather than in independent calibration and validation samples. The stability of the factor solution was not assessed using bootstrap resampling or external cross-validation. Therefore, the CFA findings should be interpreted as preliminary and require independent replication. Future psychometric studies should also examine discriminant validity, known-groups validity, and, in sufficiently diverse samples, measurement invariance across relevant caregiver groups. Finally, the psychometric evidence reported here applies to the Spanish version of the BLW-CAS; the English wording was included for reporting purposes and was not independently cross-culturally validated.

## 5. Conclusions

The Spanish BLW-CAS is a concise, multidimensional instrument with promising psychometric properties for assessing caregivers’ attitudes toward BLW. The final 16-item version showed adequate evidence of content validity and temporal stability, acceptable-to-excellent internal consistency across subscales, and a multidimensional internal structure. Subscale scores should be interpreted alongside the total score, particularly when dimension-specific conclusions are of interest. Independent replication of the factor structure in more heterogeneous caregiver samples is needed to strengthen the confirmatory evidence and support broader validation claims. By providing a BLW-specific measure of key attitudinal dimensions, the scale may facilitate more consistent research on caregiver attitudes and complementary feeding practices and may support future studies examining the relationship between caregiver attitudes and feeding behaviors or child outcomes.

## Figures and Tables

**Table 1:** Reformulation of BLW-CAS items following expert panel evaluation.

Previous Wording	Final Wording	Technical Justification
I trust my intuition and the decisions I make.	I trust the decisions I make.	The term “intuition” was removed to increase precision and avoid conceptual ambiguity.
I trust that my child is able to know when they are hungry and when they are full.	I trust that my child knows when they are hungry and when they are full.	The expression “is able to” was removed to improve linguistic economy and avoid redundancy.
I consider myself a patient person capable of giving my child the space they need when eating.	I consider myself capable of giving my child the space they need when eating.	The grammatical structure was optimized by removing non-essential personal qualifiers.
In general, I am able to tolerate a certain level of mess and/or dirtiness while my child is eating.	No changes	The original item showed adequate clarity and precision.
I am able to learn something new using the resources available to me.	I am able to learn something new related to my child’s feeding using the resources available to me.	The learning context was specified to increase construct specificity.
I am able to manage a possible choking event or emergency.	I am able to respond to a choking situation.	The term “manage” was replaced with “respond” to align with a more behavioral and situational approach.
In general, with BLW all family members eat the same food. I feel capable of changing my family’s diet to eat healthier.	I feel capable of changing my family’s diet to eat healthier, as all family members eat the same food with BLW.	The statement was restructured to improve logical cohesion between cause and consequence.
I believe that BLW promotes my child’s autonomy.	No changes	The original item showed adequate clarity and precision.
In general, I believe that BLW is an opportunity to eat as a family.	No changes	The original item showed adequate clarity and precision.
I believe that BLW fosters a positive and pleasant relationship between my child and food.	I believe that BLW fosters a positive relationship between my child and food.	The word “pleasant” was removed to increase precision and clarity.
I believe that BLW is a more natural and simpler method than others.	I believe that BLW is a more natural and/or simpler method than others.	“And/or” was introduced to allow greater interpretative flexibility.
I believe that my child is or will be ready to implement BLW.	No changes	The original item showed adequate clarity and precision.
With BLW, I believe that my child will obtain the nutrients they need for growth.	No changes	The original item showed adequate clarity and precision.
I feel capable of recognizing my child’s feeding needs and making the decisions that best benefit them.	I feel capable of making the decisions that best benefit my child based on their feeding needs.	The sentence was reorganized to improve clarity and fluency.
I feel satisfied with the information and knowledge I have about BLW.	No changes	The original item showed adequate clarity and precision.
I feel motivated and confident to start BLW.	I feel motivated for my child to follow BLW.	The focus was shifted from the caregiver to the child, consistent with the construct.
I feel that people around me respect the decisions I make regarding my child’s feeding.	No changes	The original item showed adequate clarity and precision.
I feel supported by the healthcare professionals who care for my child (pediatrician, nurse, etc.).	No changes	The original item showed adequate clarity and precision.
I maintain contact with other families and/or support groups regarding my child’s feeding.	No changes	The original item showed adequate clarity and precision.

**Table 2:** Composition of the expert panel.

Expert	Background
1	Pediatrician working in primary care. Member of the Breastfeeding Committee and the Nutrition Committee of the Spanish Association of Pediatrics.
2	Nurse with a PhD in Psychology. Holds a degree in Anthropology, with experience in questionnaire design and validation.
3	Pediatrician specialized in Pediatric Gastroenterology, Hepatology, and Nutrition. Promotes the humanization of medical care.
4	Pediatric nurse specialist. International Board-Certified Lactation Consultant (IBCLC).
5	Nurse specialist in Obstetrics and Gynecology (midwife) in primary care. Lecturer in “Child and Adolescent Nursing.”
6	Dietitian-nutritionist, lecturer, and science communicator. Has written about complementary feeding and Baby-Led Weaning.
7	Pediatric nurse specialist and lecturer. Member of the Spanish Society of Pediatric Emergency Medicine.
8	Family and Community nurse specialist with experience in questionnaire design and validation.
9	Physician specialized in Family and Community Medicine, providing care to pediatric patients in primary care. Has delivered multiple talks on healthy infant feeding and authored books on the subject.
10	Pediatrician specialized in Pediatric Gastroenterology, Hepatology, and Nutrition. Head of the pediatric nutrition unit in a tertiary hospital. Contributor to infant feeding guidelines and creator of podcasts on complementary feeding and BLW.
11	Pediatrician specialized in Pediatric Gastroenterology, Hepatology, and Nutrition. Member of the “Pediatric Nutrition” research group. Member of the Nutrition and Breastfeeding Committee of the Spanish Association of Pediatrics, the Spanish Nutrition Foundation, and the Spanish Society for Research in Pediatric Nutrition and Feeding.

**Table 3:** Test–retest descriptive statistics and reliability of the final 16-item BLW-CAS.

	N	MEAN (SD)	MEDIAN (IQR)	MIN	MAX
TEST	92	70.54 (7.95)	73 (8.25)	37	80
RETEST	92	71.28 (6.94)	72.5 (9)	38	80

Note. Descriptive statistics shown here were recalculated after item reduction using the retained 16 items. Test–retest reliability for the final 16-item version was good, with an ICC of 0.832 (95% CI: 0.757–0.886), estimated using a two-way mixed-effects model for absolute agreement, single measures. ICC = intraclass correlation coefficient; CI = confidence interval; SD = standard deviation; IQR = interquartile range.

**Table 4:** Sociodemographic characteristics of caregivers and children.

	*n*	%
**Caregiver**		
Mother	255	99.6
Father	1	0.4
**Marital status**		
Married	175	68.4
Single	79	30.8
Divorced	2	0.8
**Educational level**		
Compulsory Secondary Education	7	2.7
High school	9	3.5
University education	197	77.0
Vocational training	43	16.8
**Employment status**		
Full-time employee	130	50.8
Part-time employee	44	17.2
Self-employed	19	7.4
Unemployed	34	13.3
On leave of absence	22	8.6
Temporary disability	6	2.3
Retired	1	0.4
**Place of residence**		
Urban area	211	82.4
Rural area	45	17.6
**Number of children aged 0–24 months**		
1 child	232	90.6
2 children	23	9.0
3 or more children	1	0.4
Child age range of children aged 0–24 months (*n* = 281) *		
0–5 months	54	19.2
6–11 months	84	29.9
12–24 months	143	50.9
**Main type of feeding reported by caregiver**		
Breastfeeding	184	71.9
Formula feeding	32	12.5
Mixed feeding	40	15.6
**Experience with BLW**		
Yes	226	88.3
No	28	10.9
Not sure	2	0.8
BLW resources **		
Social media (Instagram, Facebook, Twitter, etc.)	204	79.7
Books	109	42.6
Courses	90	35.2
Breastfeeding support groups	47	18.4
Healthcare professionals	45	17.6
Academic training	43	16.8
Friends	43	16.8
Family environment	19	7.4
Work colleagues	8	3.1

Note: Percentages are based on the total number of caregivers (*N* = 256), except where otherwise indicated. * Percentages for child age range were calculated using the total number of children aged 0–24 months reported by caregivers as the denominator (*N* = 281), because some caregivers reported more than one child in this age range. ** Multiple responses were allowed; therefore, percentages do not sum to 100%.

**Table 5:** Descriptive statistics of the 19-item version administered in the validation sample according to the initial conceptual domains.

Domains and Items	Mean ± SD	95% CI
**Confidence**	**4.27 ± 0.59**	**4.19–4.34**
I trust the decisions I make.	4.42 ± 0.78	4.32–4.51
I trust that my child knows when they are hungry and when they are full.	4.30 ± 0.90	4.19–4.41
I consider myself capable of giving my child the space they need when eating.	4.29 ± 0.86	4.19–4.40
In general, I am able to tolerate a certain level of mess and/or dirtiness while my child is eating.	4.17 ± 0.86	4.07–4.28
I am able to learn something new related to my child’s feeding using the resources available to me.	4.72 ± 0.60	4.65–4.80
I am able to respond to a choking situation.	3.85 ± 0.89	3.74–3.96
I feel capable of changing my family’s diet to eat healthier, as all family members eat the same food with BLW.	4.10 ± 0.92	3.98–4.21
**Beliefs**	**4.48 ± 0.69**	**4.39–4.56**
I believe that BLW promotes my child’s autonomy.	4.82 ± 0.63	4.74–4.90
In general, I believe that BLW is an opportunity to eat as a family.	4.41 ± 0.99	4.28–4.53
I believe that BLW fosters a positive relationship between my child and food.	4.67 ± 0.72	4.58–4.76
I believe that BLW is a more natural and/or simpler method than others.	4.04 ± 1.07	3.90–4.17
I believe that my child is or will be ready to implement BLW.	4.52 ± 0.84	4.42–4.62
With BLW, I believe that my child will obtain the nutrients they need for growth.	4.42 ± 0.89	4.31–4.53
**Decision** **-** **making**	**4.46 ± 0.70**	**4.37–4.54**
I feel capable of making the decisions that best benefit my child based on their feeding needs.	4.58 ± 0.77	4.48–4.67
I feel satisfied with the information and knowledge I have about BLW.	4.25 ± 0.88	4.14–4.35
I feel motivated for my child to follow BLW.	4.55 ± 0.86	4.44–4.65
**Social interaction**	**3.25 ± 0.81**	**3.15–3.35**
I feel that people around me respect the decisions I make regarding my child’s feeding.	3.18 ± 1.10	3.04–3.31
I feel supported by the healthcare professionals who care for my child (pediatrician, nurse, etc.).	3.48 ± 1.24	3.32–3.63
I maintain contact with other families and/or support groups regarding my child’s feeding.	3.10 ± 1.43	2.92–3.27
**Total score (mean per item)**	**4.20 ± 0.53**	**4.14–4.27**

**Table 6:** Corrected item-total correlation (r.drop) and Cronbach’s alpha if the item is deleted.

Item	r.drop	α if Item Deleted
1	0.585	0.881
2	0.593	0.881
3	0.594	0.881
4	0.588	0.881
5	0.595	0.882
6	0.536	0.882
7	0.457	0.885
8	0.654	0.881
9	0.622	0.879
10	0.712	0.878
11	0.558	0.882
12	0.700	0.878
13	0.656	0.879
14	0.669	0.879
15	0.656	0.879
16	0.755	0.876
17	0.195	0.895
18	0.120	0.900
19	0.246	0.899

**Table 7:** Items with low representation in the preliminary 19-item factor solution.

Item	Content	Maximum Loading (Pattern Matrix)	Factor with Highest Loading	h^2^ (Communality)
17	I feel that people around me respect the decisions I make regarding my child’s feeding.	0.349	F2	0.113
18	I feel supported by the healthcare professionals who care for my child (pediatrician, nurse, etc.).	0.423	F2	0.135
19	I maintain contact with other families and/or support groups regarding my child’s feeding.	0.307	F3	0.130

**Table 8:** Total variance explained by the three-factor solution (16 items; polychoric matrix; PAF; oblimin rotation).

	Factor 1	Factor 2	Factor 3
SS loadings	5.83	3.17	1.79
% of total variance	36.47	19.81	11.19
Cumulative %	36.47	56.28	67.46

Note. Percentages are based on unrounded values; totals may differ by 0.01 due to rounding.

**Table 9:** Rotated pattern matrix (oblimin) and communalities from polychoric EFA for the 16-item BLW-CAS.

Item	F1 Predisposition	F2 Self-Efficacy	F3 Practical Skills	h^2^
**i8**	**0.974**			**0.944**
**i11**	**0.853**			**0.644**
**i9**	**0.848**			**0.752**
**i10**	**0.793**			**0.766**
**i16**	**0.786**			**0.851**
**i13**	**0.771**			**0.724**
**i12**	**0.755**			**0.725**
**i15**	**0.475**		0.318	**0.581**
**i3**		**0.821**		**0.751**
**i1**		**0.746**		**0.639**
**i14**		**0.715**		**0.759**
**i2**	0.315	**0.546**		**0.545**
**i7**			**0.609**	**0.458**
**i5**		0.300	**0.580**	**0.624**
**i4**		0.316	**0.492**	**0.561**
**i6**			**0.433**	**0.470**

Note. Loadings with absolute values <0.30 are omitted for readability. Bold values indicate the primary loading used for factor assignment; loadings ≥0.40 were considered salient. h^2^ = communality.

**Table 10:** Internal consistency and descriptive statistics of the final 16-item BLW-CAS.

Scale	No. of Items	Cronbach’s α	McDonald’s ω	Mean	SD
F1 Predisposition	8	0.913	0.953	4.46	0.68
F2 Self-efficacy	4	0.830	0.883	4.40	0.68
F3 Practical skills	4	0.757	0.807	4.21	0.63
Total (16 items)	16	0.923	0.948	4.38	0.58

Note. Means and standard deviations are expressed as item-level scores ranging from 1 to 5. Cronbach’s α was calculated from the observed item scores, whereas McDonald’s ω was estimated from the polychoric correlation matrix. Both coefficients are reported as internal consistency estimates for the final 16-item version.

**Table 11:** Correlations between BLW-CAS subscales (*N* = 256). Spearman’s ρ.

Subscale	F1 Predisposition	F2 Self-Efficacy	F3 Practical Skills
F1 Predisposition	1.000	0.585	0.510
F2 Self-efficacy	0.585	1.000	0.538
F3 Practical skills	0.510	0.538	1.000

Note: All correlations were statistically significant (*p* < 0.001).

**Table 12:** Standardized factor loadings from CFA (three-factor model, 16 items; WLSMV; *N* = 256).

Factor 1 (Predisposition)	λ (std.)	Factor 2 (Self-Efficacy)	λ (std.)	Factor 3 (Practical Skills)	λ (std.)
i8	0.943	i14	0.909	i5	0.784
i16	0.934	i3	0.798	i4	0.775
i10	0.895	i2	0.770	i6	0.730
i12	0.866	i1	0.762	i7	0.591
i13	0.840				
i9	0.827				
i15	0.766				
i11	0.746				

Note: λ (std.) = standardized factor loading. All loadings were moderate to high (λ = 0.591–0.943).

**Table 13:** Convergent validity of the BLW-CAS with external reference measures (Pearson correlations; *N* = 256).

Comparison	r	95% CI	*p* Value	*p* Value (Holm)
BLW-CAS vs. General self-efficacy	0.298	[0.182; 0.406]	<0.001	<0.001
BLW-CAS vs. Decision-making satisfaction	0.350	[0.238; 0.453]	<0.001	<0.001

Note: r = Pearson’s correlation coefficient; 95% CI = 95% confidence interval; *p*-value (Holm) = *p*-value adjusted for multiple comparisons using the Holm method.

## Data Availability

The data presented in this study are available on request from the corresponding author due to privacy.

## Supplementary Materials

The following supporting information can be downloaded at: https://www.mdpi.com/article/10.3390/healthcare14142214/s1, Supplementary File S1. Spanish BLW-CAS and English wording for reporting purposes.
