# Supplementary material for: Caregivers’ Attitudes Toward Baby-Led Weaning: Development and Psychometric Validation of the BLW-CAS
Source: Healthcare (Basel). 2026 Jul 21;14(14):2214. doi: 10.3390/healthcare14142214 (PMC13409823; doi:10.3390/healthcare14142214)
Supplement: Supplementary file 1 [file healthcare-14-02214-s001.zip › healthcare-4335931-supplementary.pdf]

Escala de actitudes hacia el Baby-Led Weaning en cuidadores

(BLW Caregiver Attitudes Scale; BLW-CAS versión en español)

Opciones de respuesta: 1 = Totalmente en desacuerdo; 2 = En desacuerdo; 3 = Ni de acuerdo ni en desacuerdo; 4 = De acuerdo; 5 = Totalmente de acuerdo

1. Confío en las decisiones que tomo.
2. Confío en que mi hijo/a sabe cuándo tiene hambre y cuándo está saciado/a.
3. Me considero capaz de dar a mi hijo/a el espacio que necesita cuando come.
4. En general, soy capaz de tolerar un cierto desorden y/o suciedad mientras mi hijo/a come.
5. Soy capaz de aprender algo nuevo relacionado con la alimentación de mi hijo/a empleando los medios que tengo a mi alcance.
6. Soy capaz de reaccionar ante una situación de atragantamiento.
7. Me siento capaz de cambiar la alimentación de mi familia para comer más saludable, ya que con el BLW todos los miembros comen la misma comida.
8. Considero que el BLW promueve la autonomía de mi hijo/a.
9. En general, considero que el BLW es una oportunidad para comer en familia.
10. Considero que el BLW fomenta una relación positiva de mi hijo/a con la comida.
11. Considero que el BLW es un método más natural y/o sencillo que otros.
12. Considero que mi hijo/a está o estará preparado/a para poner en práctica el BLW.
13. Con el BLW considero que mi hijo/a tendrá los nutrientes que necesita para su crecimiento.
14. Me siento capaz de tomar las decisiones que más beneficien a mi hijo/a en función de sus necesidades alimentarias.
15. Me siento satisfecho/a con la información y los conocimientos que tengo sobre el BLW.
16. Me siento motivado/a para que mi hijo/a siga el BLW.

Nota. *El cuestionario final consta de tres dimensiones: predisposición hacia el BLW (ítems 8, 9, 10, 11, 12, 13, 15 y 16), autoeficacia en la alimentación (ítems 1, 2, 3 y 14) y habilidades prácticas (ítems 4, 5, 6 y 7). La puntuación total oscila entre 16 y 80 puntos, con puntuaciones más altas indicando actitudes más favorables hacia el BLW. También pueden calcularse puntuaciones medias por ítem para la escala total y para cada dimensión. La versión en inglés se proporciona únicamente con fines de presentación y no ha sido sometida a un proceso de adaptación transcultural.*

Questionnaire on Caregivers' Attitudes toward Baby-Led Weaning (English version)

Response options: 1 = Strongly disagree; 2 = Disagree; 3 = Neither agree nor disagree; 4 = Agree; 5 = Strongly agree.

1. I trust the decisions I make.
2. I trust that my child knows when they are hungry and when they are full.
3. I consider myself capable of giving my child the space they need when eating.
4. In general, I am able to tolerate a certain level of mess and/or dirtiness while my child is eating.
5. I am able to learn something new related to my child's feeding using the resources available to me.
6. I am able to respond to a choking situation.
7. I feel capable of changing my family's diet to eat healthier, as all family members eat the same food with BLW.
8. I believe that BLW promotes my child's autonomy.
9. In general, I believe that BLW is an opportunity to eat as a family.
10. I believe that BLW fosters a positive relationship between my child and food.
11. I believe that BLW is a more natural and/or simpler method than others.
12. I believe that my child is or will be ready to implement BLW.
13. With BLW, I believe that my child will obtain the nutrients they need for growth.
14. I feel capable of making the decisions that best benefit my child based on their feeding needs.
15. I feel satisfied with the information and knowledge I have about BLW.
16. I feel motivated for my child to follow BLW.

***Note**. The final questionnaire comprises three dimensions: predisposition toward BLW (items 8, 9, 10, 11, 12, 13, 15, and 16), self-efficacy in feeding decisions (items 1, 2, 3, and 14), and practical skills (items 4, 5, 6, and 7). The total score ranges from 16 to 80, with higher scores indicating more favorable attitudes toward BLW. Mean item scores may also be calculated for the total scale and each dimension. The English wording is provided for reporting purposes only and has not been cross-culturally validated.*
